# General principles for the classification of analysers

**DOI:** 10.1155/S1463924688000331

**Published:** 1988

**Authors:** R. Haeckel

**Affiliations:** Zentralkrankenhaus Inst. für Laboratoriumsmedizin Bremen 1 D2800 Germany

## Abstract

The IUPAC Commission on Automation and New Technologies has developed a scheme for the classification of analytical systems. The classification scheme is reported in this paper.


Journal of Automatic Chemistry Vol. 10, No. 4 (October–December 1988), pp. 164–166

### General principles for the classification of analysers

**R. Haeckel**

*Zentralkrankenhaus, Inst. für Laboratoriumsmedizin, D2800 Bremen 1, FR Germany*

*The IUPAC Commission on Automation and New Technologies has developed a scheme for the classification of analytical systems. The classification scheme is reported in this paper.*

### Introduction

The classification scheme suggested by IUPAC's Commission on Automation and New Technologies should be helpful in making the various concepts of mechanized and automated analytical systems understandable. It should be used as a guide for teaching automation in clinical chemistry, and as a base for describing new instrumentation.

For definition of terms the reader is referred to references [1] and [2]. A mechanized analytical system consists of an analyser and the reagents required to perform a specific analysis. The term 'analyser' is similar to a measuring system which is defined in metrology [3] as 'a complete set of measuring instruments and another equipment assembled to carry out a specified measured task'.

Analytical systems are generally classified in terms of their type of transport system, for example discrete and flow procedures (see table 1).

Further subdivisions take into account methodology, number of tests, sampling and processing procedures or test selection.

*Table 1. Classification of analysers in clinical chemistry. (D indicates discriminate, or selective, mode and ID indicates indiscriminate, non-selective mode.)*

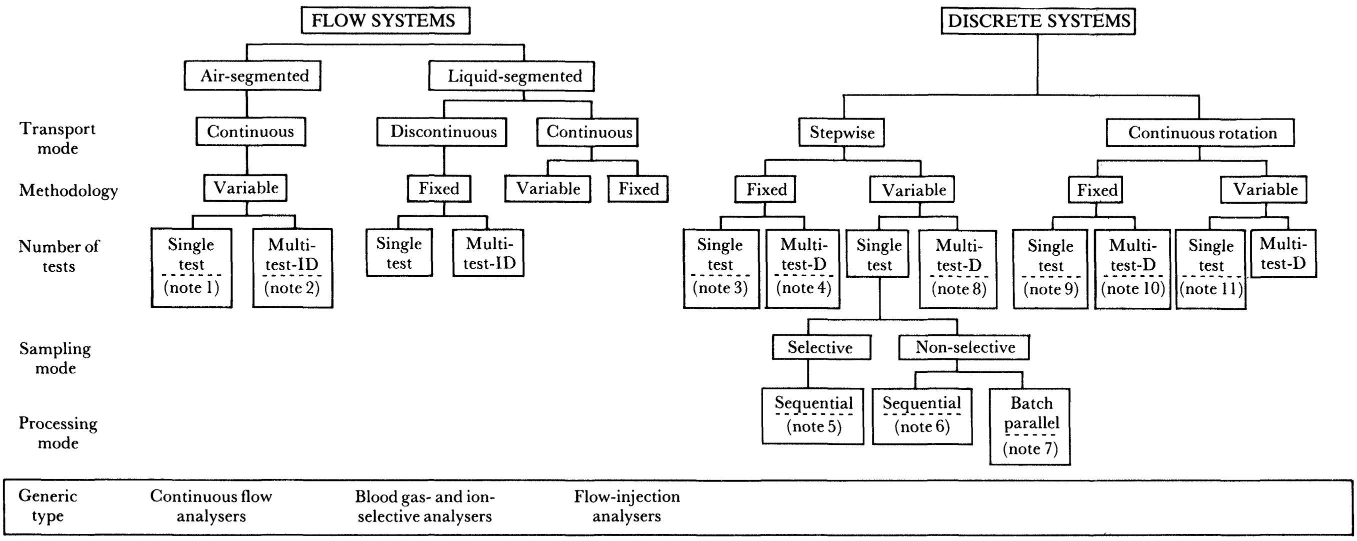

Examples of analysers which are (or have been) available on the market:

Note 1 AutoAnalyzer*, STASAR. * Air and liquid segmented.

Note 2 SMA*, SMAC*, Chem-1.

Note 3 Creatinine analyser, Glucose analyser, BUN-analyser, Reflolux.

Note 4 Ektachem, Seralyzer, Paramax, Aca, Astra 4/8, IL 919, KLiNa, AFM 5051, Stratus, Reflotron.

Note 5 XYP-analyser, Epos, Mira.

Note 6 Quantachem, ACP 5040, ABA 100/200, VP, Kem-o-Mat, PA 800, System 3500, Impact 400, Megalyzer.

Note 7 Kone CD, FP 9.

Note 8 G 400, Hitachi 706, Hitachi 712, Hitachi 737, Parallel, Hycel M, GSA 11, RA 1000, Eris, DACOS, Kone Progress.

Note 9 Gemini.

Note 10 SAMEA.

Note 11 Flexigem, Cobas-Bio, Multistat 111, Centrifichem, Fara.

*Table 2. Classification of analytical systems.*

| | |
|---|---|
| 1 | Flow-systems |
| 1.1 | Gas-segmented systems |
| 1.2 | Liquid-segmented systems |
| 1.2.1 | Continuous systems |
| 1.2.2 | Discontinuous systems |
| 2 | Discrete systems |
| 2.1 | Stepwise-transport |
| 2.1.1 | With fixed methodology |
| 2.1.1.1 | Single-test analysers with reagents in solution |
| 2.1.1.2 | Multitest analysers with prepacked reagents |
| 2.1.1.2.1 | Analytical systems with carrier-bound reagents (one-layer techniques, multi-layer techniques) |
| 2.1.1.2.2 | Reagents in tablets |
| 2.1.1.2.3 | Reagents in foils |
| 2.1.2 | With variable methodology |
| 2.1.2.1 | Single-test systems |
| 2.1.2.1.1 | With selective sampling (random access) |
| 2.1.2.1.2 | With non-selective sampling |
| 2.1.2.2 | Multi-test systems |
| 2.2 | Centrifugal systems |

In table 2 numbering of the various classes is suggested. Group 1 can be subdivided in a similar way to group 2 in table 1; however, this subclassification has little practical relevance in today's instrumentation market-place.

### Flow systems

Flow systems can be subdivided according to:

(1) The interruption of the stream by gas-segments, liquid-segments or both.
(2) Transport rhythm – continuous or discontinuous systems.
(3) The way the specimen is introduced to the system – aspiration or injection.

The only gas which has been used so far to separate various segments is air. The best known example of an air-segment is the Autoanalyzer (Technicon Instruments Corporation).

The presence of air has been considered essential for preventing sample dispersion with consequent carry-over in continuous flow analysers. Stewart *et al.* [4] and Ruzicka and Hanson [5 and 6], however, have shown that air bubbles can be omitted if the sample is introduced by means of injection (flow injection systems) and if the inner diameter of the analytical conduits is reduced. In these systems segments of various liquids are usually encountered.

### Discontinuous systems

In discontinuous flow systems (also called intermittent systems) each sample is analysed in one cycle, during which sample and reagents continuously flow through the analytical and detecting system. Each cycle consists of sample uptake, analysis, display of result, and, in some cases, rinsing the system. After completion of the cycle, the system stops and waits until the next cycle is initiated. Examples are blood gas and ion-selective analysers.

### Discrete systems

The discrete systems represent a heterogeneous group which includes many different concepts. Their only common characteristic is discrete sample processing which involves transport of the sample or reaction mixture within an individual container. In some analysers the chemical reactions are first performed in a discrete mode, and then the reaction mixture is discontinuously transferred into a flow cell to measure the signal obtained (combination of discrete sample processing with discontinuous flow procedure for signal detection). In some systems, the method cannot be altered by the user (fixed methodology), whereas in others the methods can be changed (variable methodology).

The transport or transfer of the materials is either performed in steps or continuously by centrifugal force. In contrast to flow systems, discrete analysers more or less imitate manual procedures. All sample processing steps can also be performed with manual pipetting devices.

### *Discrete systems with fixed methodology*

Single-test systems determine only one analyte, in contrast to multitest systems which determine several analytes per specimen. For example, various analysers are available that measure glucose only using the glucose oxidase reaction, the hexokinase reaction or the strip methodology. Discrete single-test analysers are particularly well suited for emergency laboratories, intensive care units and specialized out-patients unit.

Multitest analytical systems (under 2.1.1.2) comprise prepacked reagent systems (see table 2).

Analytical systems with carrier-bound reagents can be subdivided into two groups:

(1) One-layer techniques usually apply a strip of paper, or another absorbent material, containing the reagents necessary for the qualitative or quantitative measurement of an analyte. A transducer is used to follow the reaction by measuring the light reflected from the surface of the strip on which the sample is applied.

(2) Multilayer techniques are composed of multiple layers made from a gelatin or polymer matrix held in a plastic slide mount. Each layer may contain a separate reagent, allowing the reaction to proceed sequentially. A transducer is used to follow the reaction by measuring the light reflected from one of the layers after transmission of the light through the reaction layers. The measurement of electrolytes has been made possible by an ion selective electrode system comprising two identical electrodes linked by a bridge and held in a plastic slide mount. The potential difference produced between the reference solution placed upon one electrode and a patient sample placed on the other electrode is used to calculate, by the Nernst equation, the ionic concentration in the sample.

An example of an analytical system using reagents in tablets is the Paramax from Merz and Dade; the ACA from Dupont is an example using reagents prepacked in foil.

### Discrete systems with variable methodology

In single-test analytical systems with variable methodology the user has a choice of reagents source, and, in some cases, or the method applied for a particular analyte. After one run has been completed, the analyser can be prepared for another method (single-test systems with non-selective sampling). In a recent generation of analysers selective sampling from each batch is possible: various batches are processed sequentially but samples are taken only from those specimens on which the particular test is requested.

Multitest analytical systems in this group are selective – the operator can select which test(s) per specimen will be performed (the term 'random access' is incorrect for these machines). Examples are the Parallel, the Prisma and the Hitachi 737.

Multitest analysers with less capacity (about 150–500 tests per hour) are the G 450 (Greiner Electronics), Hitachi 705, Technicon RA 1000 and ERIS (Olympus). The Astra 8 (Beckman Instruments) uses fixed and variable methodologies.

### Centrifugal systems

Centrifugal systems apply the centrifugal force for the transport of samples and reagents.

The cuvette-rotating principle has been reversed so that the light beam rotates rather than the cuvettes. Therefore, sampling can be performed continuously and a transfer rotor is unnecessary. The first system has been introduced by Coulter Electronics, called Dacos. Since the centrifugal force is not applied in this system it is allocated to group 2.1.2.2 in table 2.

### References

1. Hjelm, M., Bierens de Haan, J., Büttner, J. and Young, D. S., Recommended nomenclature for automated analysis. *IUPAC Information Bulletin*, **3** (1978), 233–240.
2. Haeckel, R., Mechanizatrion and automation of analysis. In H. U. Bergmeyer (Ed) *Methods of Enzymatic Analysis*, 3rd edn, Vol. I (Verlag Chemie, Weinheim, 1984), 450.
3. *International Vocabulary of Basic and General Terms in Metrology* (International Organization for Standardization, Geneva, 1984).
4. Stewart, K. K., Beecher, G. R. and Hare, P. E., Rapid analysis of discrete samples: the use of nonsegmented, continuous flow. *Analytical Biochemistry*, **70** (1976), 167–173.
5. Ruzicka, J. and Hansen, E. H., Flow injection analysis, Part I. A new concept of fast continuous flow analysis. *Analytica Chimica Acta*, **78** (1975), 145–157.
6. Ruzicka, J. and Hansen, E. H., Flow injection analysis. Principles, applications and trends, *Analytica Chimica Acta*, **114** (180), 19–44.

### MAS

The Manufacturing Advisory Service (MAS) has recently been launched by the Leatherhead Food R.A. (LFRA). Arising out of a consultancy service established over the last six years, the MAS will draw on the extensive scientific, technical and information resources of the world's largest food research association.

The role of MAS is to help companies improve their techniques of factory management and thereby achieve ever higher standards of quality. MAS does this by assisting in the spread of new technology and by providing consultancy advice in all aspects of general food manufacture in a production environment. First-line advice can be provided on such subjects as factory design and plant layout, equipment selection, factory services, energy management, factory operation, and planning and control of manufacturing operations.

*Further information from Richard Swift, Sales Manager, The Leatherhead Food R.A., Randalls Road, Leatherhead, Surrey KT22 7RY, UK. Tel.: 0372 376761.*

